# Bathymetric distribution of parasitic copepods: strategies for adaptation to the deep-sea environment

**DOI:** 10.1017/S0031182025100851

**Published:** 2025-10

**Authors:** Nanami Yumura, Susumu Ohtsuka, Ryo Misawa, Shigeaki Kojima

**Affiliations:** 1 Atmosphere and Ocean Research Institute, The University of Tokyo, Kashiwa, Japan; 2 Fisheries Laboratory, Blue Innovation Division, Seto Inland Sea Carbon-Neutral Research Center, Hiroshima University, Takehara, Japan; 3 Demersal Fish Resources Division, Fisheries Stock Assessment Center, Fisheries Resources Institute, Japan Fisheries Research and Education Agency, Hachinohe, Japan; 4 National Fisheries University, Japan Fisheries Research and Education Agency, Shimonoseki, Japan

**Keywords:** adaptation, bathymetric distribution, deep-sea fish, parasitic copepod, Western Pacific

## Abstract

The bathymetric distribution and species richness of marine parasites are generally influenced by host-related and environmental factors. While parasite traits such as attachment modes and reproduction strategies are believed to play important roles in shaping these patterns, insights into the influence of these traits remain limited. To enhance our understanding regarding the bathymetric distribution of deep-sea parasites and the biological traits associated with successful colonization of deep-sea habitats, we compiled occurrence data on parasitic copepods parasitizing deep-sea fishes, based on both current and previous records. We found that species richness declined with increasing depth, likely reflecting host distribution patterns. The recorded maximum depths of copepods in the families Chondracanthidae, Lernaeopodidae, Pennellidae and Sphyriidae exceeded 2000 m. These families are characterized by the following traits: suitable attachment sites like gills for efficient nutrient intake; firm attachment modes with limited mobility that enable efficient energy use; reproductive strategies such as the presence of dwarf males or the use of intermediate hosts; and low host specificity. Among all copepods parasitizing fish, a chondracanthid *Chondracanthodes deflexus* Wilson, 1932 had the deepest occurrence record and was the only species found in the abyssal region (>4000 m). This species exhibited a relatively high intensity (9.6), possibly because of the challenges of locating hosts in an environment with extremely low host density. These results indicate that the colonization of deeper waters by parasitic copepods may have proceeded via a stepwise process involving both the retention and acquisition of traits advantageous for survival under increasingly extreme conditions.

## Graphical abstract

**Figure:**
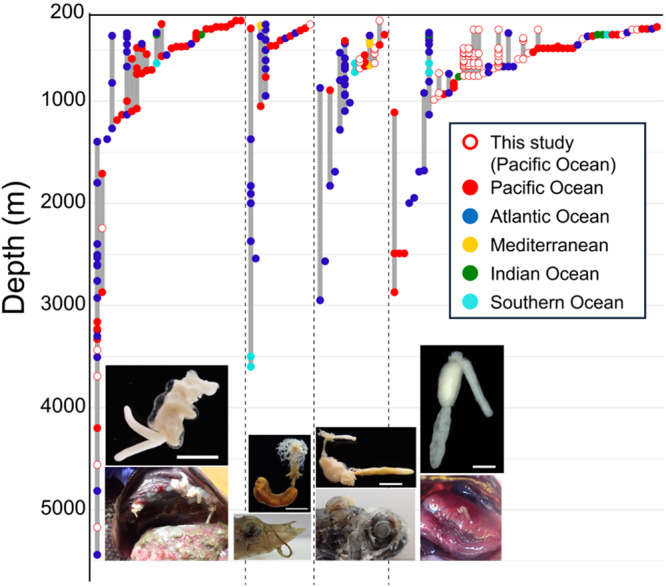

## Introduction

The distribution and species richness of deep-sea parasites are influenced by host availability and environmental factors (Rohde 1984; Poulin and Rohde 1997). Generally, in the case of ectoparasites, biological and environmental factors play critical roles in determining their bathymetric distribution (Rohde 1993). Parasites encompass a wide range of taxa with diverse traits, including differences in modes of attachment, reproduction strategies, life history patterns and host specificity. Although host distribution is generally considered the primary determinant of parasite distribution, Poulin (2021) has highlighted that such parasitic traits may also contribute to determining parasites’ distribution. However, despite a growing recognition of the potential significance of the biological traits of parasites, relatively few studies have examined their role in determining bathymetric distribution (Llopis-Belenguer et al. 2019).

To address this gap, in this study, we focused on parasitic copepods, a group known for their diverse morphological features and ecology. Parasitic copepods, particularly those that parasitize fish, tend to have specialized morphologies for reproduction and host attachment, with body sizes differing according to taxon and host species (Kabata 1979). Although the mode of attachment is generally ectoparasitic, some species are mesoparasitic, in which the anterior part of the body (with the largest number of somites) is embedded within the host tissues and the posterior part remains exposed to water (Kabata 1976; Piasecki et al. 2022). The reproductive modes of these parasites also differ, with some utilizing intermediate hosts and others having so-called ‘dwarf’ males resulting from arrested development (Boxshall and Halsey 2004). Although a few families of siphonostomatoid and cyclopoid species have been reported in deep-sea fishes (Boxshall 1989; Ho 1993), the majority tend to be parasitic on shallow-water hosts, with only a few genera colonizing the deep sea and only two families considered specialists in deep-sea fishes (Boxshall 1998). However, most of the studies conducted to date on deep-sea parasitic copepods have been descriptive, with only a few focusing on ecological aspects, such as host specificity and bathymetric distribution (Boxshall 1998; Cañás et al. 2013).

In this study, we summarize information regarding the depth records of parasitic copepods infecting deep-sea fishes based on current and previous records to determine their bathymetric trends. Moreover, to assess the influence of depth on parasite occurrence, the prevalence and intensity of these parasites were determined for different depth ranges and their patterns were analysed. Furthermore, we examined the contribution of specific biological traits to the colonization of deeper environments, thereby providing insights into the processes underlying successful deep-sea invasion. To reveal the ecological shifts from shallower depths with large environmental changes to more stable deeper depth conditions, in this study deep water was defined as the layer below 200 m depth in this study.

## Materials and methods

### Specimens

Deep-sea fish samples (≥200 m) were collected by purchasing specimens from vendors and sampling using deep-sea trawls during cruises of the Research vessels *Hakuho-maru* (KH-23-5) and *Wakataka-maru* between 2018 and 2024. In addition, we examined specimens of deep-sea fish, particularly those of demersal fishes, such as macrourids, preserved at the National Museum of Nature and Science, Tsukuba, Japan, and the Atmosphere and Ocean Research Institute, The University of Tokyo. These specimens were collected between 1968 and 2023 and fixed in 10% neutral formalin and/or preserved in 99.5% ethanol. Macrourids were prioritized given their wide bathymetric distribution (Merrett et al. 1991) and the fact that a high diversity of parasitic copepods has been observed infecting these fish (Boxshall 1998).

**Figure 1.**
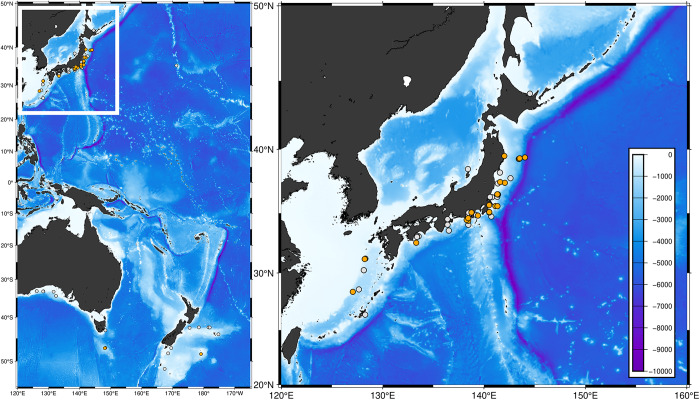
Map of the sampling sites used in this study. Orange circles indicate the sites at which parasitic copepods were obtained. Gray circles indicate the sites at which no parasitic copepods were obtained. This map was produced using GMT 6 (Wessel et al. 2019) based on bathymetry data from ETOPO1 (Amante and Eakins 2009).

A total of 2154 fish specimens, obtained from depths of between 200 and 5171 m in the Western Pacific Ocean, including Japanese, Australian and New Zealand waters, were used in this study (Figure 1; Table S1). Host fish were identified based on their morphological features according to Nakabo (2013) and Roberts et al. (2015). Following identification, external surfaces such as fins, gill cavities and mouths were carefully examined with the naked eye to identify ectoparasitic and mesoparasitic copepods. When parasites were observed, they were removed using dissecting needles and fine forceps and preserved in 99.5% ethanol. The copepods thus obtained were identified based on their morphological features according to published descriptions (Yamaguti 1939; Shiino 1955, 1956; Ho and Kim 1989; Ho 1993, 1994; Cheng et al. 2022; Izawa 2022). Morphological terminology of copepods follows that of Huys and Boxshall (1991).

### Literature survey

A total of 265 records of parasitic copepods parasitizing deep-sea fishes (≥200 m) were compiled from 69 papers published between 1886 and 2024 (see Tables S2 and S3 for detailed information on the records). The taxonomic nomenclature of the parasites and their hosts followed the World of Copepods database (Walter and Boxshall 2025) and FishBase (Froese and Pauly 2025), respectively, which were used as references for the currently accepted classifications. Records lacking species-level identification, and those records associated with phylogenetically unstable lineages, were excluded from the analysis, as were records with uncertain depth information or those considered to reflect bycatch specimens captured during net winding. For records with large gaps between coordinates and depths, the median values of the initial and final sampling points were used.

### Patterns of bathymetric distribution

The bathymetric distribution of parasitic copepods was analysed using combined datasets obtained in the present and previous studies. The numbers of families and species of parasitic copepods were determined for 100-m depth intervals within each ocean to examine changes in species richness across depth ranges and to compare these changes between the Pacific and Atlantic Oceans. The suitability of species richness and sampling effort (number of records) was tested by creating species accumulation curves (SACs). This analysis was performed using the R software package vegan v. 2.7 (Oksanen et al. 2008). To statistically evaluate the similarity in species richness patterns between hosts and parasites across various depths, Spearman’s rank correlation analysis was applied to the species count data for each depth interval using R software 4.3.1 (R Development Core Team 2023), with a significance threshold of *P* <0.05. Host species data were obtained from Nakayama (2022), Smith and Brown (2002) and Priede et al. (2010).

For each species and individual sampling effort, we determined prevalence, which represents the percentage of fish specimens infected with parasitic copepods (calculated by dividing the number of infected hosts by the number of hosts examined), and mean intensity, which is the average number of individuals of a specific parasitic copepod species infecting a single infected host (calculated by dividing the total number of parasitic copepods by the number of infected hosts) (Bush et al. 1997). These determinations were based solely on data obtained from our examination of specimens, including both preserved and newly collected material. These values were calculated on a per-site (per-net) basis, regardless of whether the specimens were sourced through museum surveys or new sampling.

## Results

**Figure 2.**
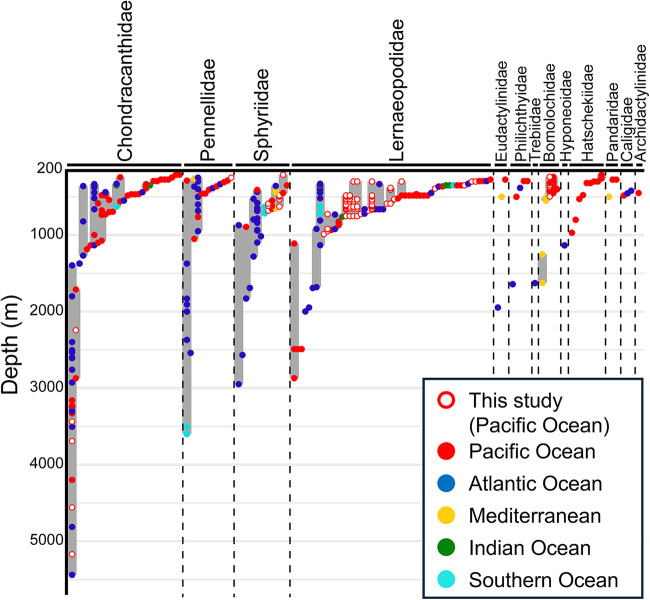
Bathymetrical distribution of copepods parasitic on deep-sea fishes. Species are listed in Supplementary Information 1.

During specimen surveys, we obtained 1190 parasitic copepod specimens from 246 hosts in the Western Pacific Ocean (Figure 1). These copepods were classified into 5 families, 14 genera and 28 species, whereas the hosts were members of seven families, 11 genera, and 19 species (Table S1). On the basis of our specimen examination and literature survey, we identified a total of 13 families, 63 genera and 150 species of parasitic copepods infecting deep-sea fishes (>200 m) worldwide (Figure 2; Table S1).

**Figure 3.**
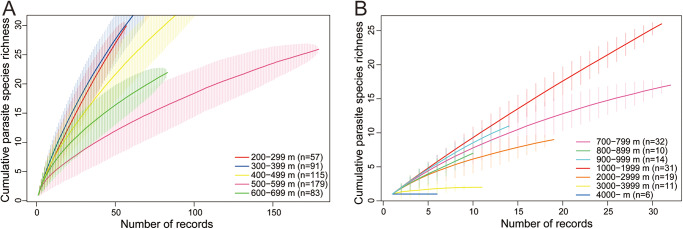
Species accumulation curves with the number of records for each depth interval. (A) Species accumulation curves between depths of 200 and 700 m. (B) Species accumulation curves below depths of 700 m.

SACs for shallower depths (200–699 m) revealed that the number of parasitic copepod species increased rapidly with the sampling effort and did not tend towards a plateau (Figure 3A). In contrast, SACs for deeper depths (>700 m) showed much lower species richness, with curves tending to plateau at low species counts, indicating limited species accumulation, even with additional records (Figure 3B).

**Figure 4.**
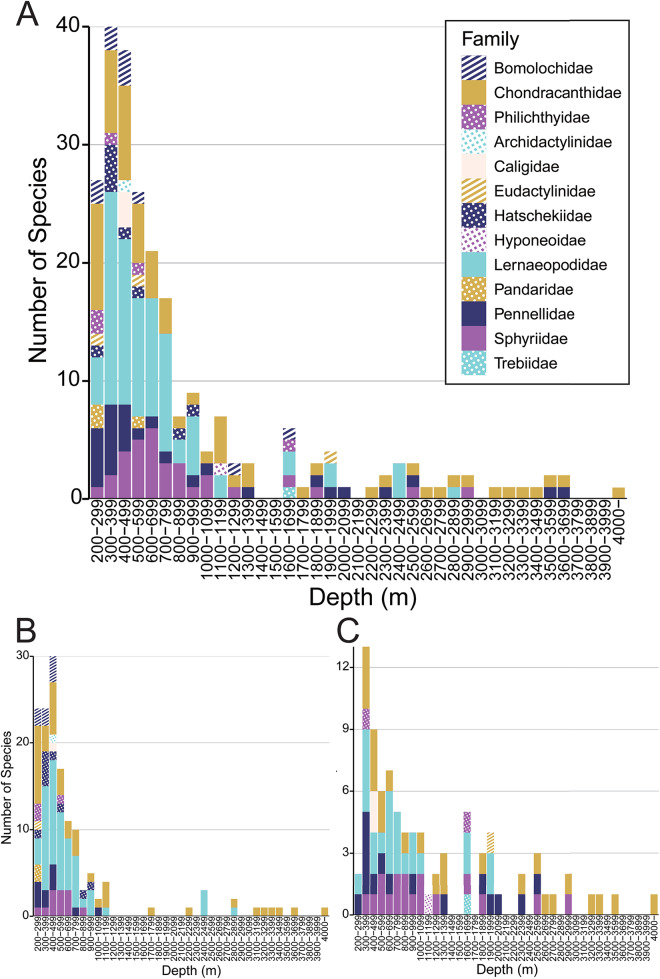
Species richness at 100-m depth intervals. (A) Data for the number of families and species data across all ocean areas. (B) Data from the Pacific Ocean. (C) Data from the Atlantic Ocean.

Based on the combined results of the specimen and literature surveys, 93% of the species were recorded at depths <1000 m, and 71% of the species that were distributed at depths >2000 m belonged to the families Chondracanthidae, Pennellidae, Sphyriidae and Lernaeopodidae. Only a single species of each of the two families Chondracanthidae and Pennellidae – *Chondracanthodes deflexus* and *Sarcotretes eristaliformis* (Brian 1908), respectively – was recorded at depths >3000 m. Moreover, only the former was shown to inhabit depths >4000 m (Figure 2). Species richness was highest between the depths of 300 and 399 m, and thereafter gradually declined with increasing depth (Figure 4A). In the Pacific Ocean, we detected a sharp decline in species richness at depths from 400 to 2000 m (Figure 4B), whereas in the Atlantic Ocean, the peak of species richness was identified within the depth range 300 to 399 m, with a subsequent gradual decline to a depth of 3000 m (Figure 4C). The bathymetrical changes in parasite species richness across depth intervals (the present Pacific dataset) exhibited a highly significant correlation with those of host species (grenadiers) recorded around Japan by Nakayama (2022) (Spearman’s ρ = 0.80, *P* < 0.001). A moderate but statistically significant positive correlation was also shown across depth intervals in pelagic fishes in the Northeastern Pacific Ocean, based on Smith and Brown (2002) and the present Pacific parasite dataset (Spearman’s ρ = 0.55, *P* < 0.001). Additionally, a significant positive correlation was observed between demersal fish in the Atlantic, based on Priede et al. (2010) and the present Atlantic dataset (Spearman’s ρ = 0.33, *P* = 0.02).

**Figure 5.**
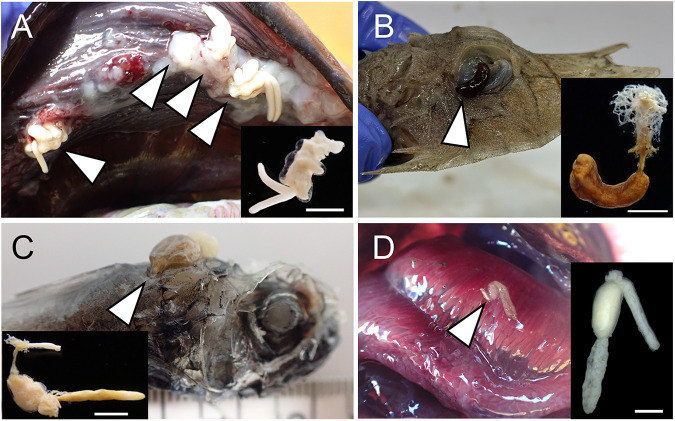
Photographs of copepods parasitic on deep-sea fishes, showing their attachment to the host and whole-body morphology. (A) *Chondracanhodes deflexus* (Chondracanthidae) attached to the gill cover of *Coryphaenoides yaquinae*. (B) *Phrixocephalus* sp. (Pennellidae) embedded in the eye of *Heminodus philippinus*. (C) *Lophoura cardusa* (Sphyriidae) embedded near the base of the dorsal fin of *Hymenocephalus striatissimus*. (D) *Clavella collaris* (Lernaeopodidae) attached to the gill of *Coelorinchus gilberti*. Scale bars: A, B and C, 5 mm; D, 1 mm.

Among the identified chondracanthid species, 70% were recorded at depths <500 m, whereas 29% were recorded at depths >1000 m (Figure 2). These species typically attach to hosts using their antennae, and in some cases, the head becomes embedded within the host tissues (Figure 5A). Among all copepods parasitizing fish, the deepest record was obtained for *Chondracanthodes deflexus*, which was collected at a depth of 5440 m (Boxshall 1998). Off Tohoku (northeastern Japan), the species was found at depths of between 3436 and 5171 m, with a prevalence of 50% and a mean intensity of 9.6 (range, 1–22) (Table 1). Similar to shallow-water chondracanthids, the hosts of deep-sea chondracanthids are demersal fish in which the gills are the primary site of parasite attachment (Tables 1, S1 and S2; Boxshall and Halsey 2004).

**Table 1.** Information on parasitic copepods obtained from our specimen examination at sampling sites where parasitic copepods were found and at least 10 fish which copepods could be parasitic on were obtained except for *Chondracanthodes deflexus*

	Locality	Date	Depth	Prevalence	Intensity	Intensity range	Num. host	Attachment site
**Bomolochidae**								
*Hamaticolax coelorinchi* from *Coelorinchus kishinouyei*								
	Suruga Bay	1969	NA	0.46	6.83	1–23	13	Gill
	Suruga Bay	17 Nov 1993	370	0.76	2.84	1–5	25	Gill
	Suruga Bay	17 Jan 2023	240	0.51	4	1–21	43	Gill
**Chondracanthidae**								
*Chondracanthodes deflexus* from *Coryphaenoides yaquinae*								
	Japan Trench	26 Sep 2023	5171	0.25	1	1	4	Gill
	Japan Trench	27 Sep 2023	3436	0.5	22	22	2	Gill
	Japan Trench	27 Sep 2023	3692	1	14	14	1	Gill
	Japan Trench	27 Sep 2023	4559	0.67	5.5	3–8	3	Gill
	All site in Japan Trench	26–27 Sep 2023	3436–5171	0.5	9.6	1–22	10	Gill
**Lernaeopodidae**								
*Clavella collaris* from *Coelorinchus gilberti*								
	Fukushima	09 Nov 2023	653	0.95	5.1	1–11	21	Gill
	Fukushima	14 Nov 2023	496	0.33	1.3	1–3	18	Gill
	Fukushima	14 Nov 2023	533	0.75	3.3	1–9	56	Gill
	Fukushima	15 Nov 2023	482	0.95	2.9	1–7	20	Gill
	Fukushima	15 Nov 2023	559	1	7.4	1–25	20	Gill
*Clavella diversia* from *Coelorinchus gilberti*								
	Fukushima	09 Nov 2023	653	0.57	1.75	1–3	21	Gill
	Fukushima	14 Nov 2023	496	0.5	1.33	1–3	18	Gill
	Fukushima	14 Nov 2023	533	0.7	2.46	1–8	56	Gill
	Fukushima	15 Nov 2023	482	0.95	2.05	1–4	20	Gill
	Fukushima	15 Nov 2023	559	0.9	3.11	1–7	20	Gill
*Clavella sokodara* from *Coryphaenoides nasutus*								
	Chiba	22 June 2001	527	0	0	0	18	Gil
	Kumano-nada	26 May 2002	821	0	0	0	11	Gil
*Clavella* sp. 1 from *Coelorinchus gilberti*								
	Fukushima	09 Nov 2023	653	0.1	1	1	21	Mouth
	Fukushima	14 Nov 2023	496	0	0	0	18	Mouth
	Fukushima	14 Nov 2023	533	0.23	1.31	1–2	56	Mouth
	Fukushima	15 Nov 2023	482	0.05	1	1	20	Mouth
	Fukushima	15 Nov 2023	559	0.3	1.33	1–2	20	Mouth
*Clavella* sp. 4 from *Coelorinchus innotabilis*								
	New Zealand	14 Nov 1983	726	0.07	2	2	14	Gill
	New Zealand	NA	NA	0	0	0	10	Gill
*Clavella* sp. 5 from *Coelorinchus japonicus*								
	Kochi	11 Sep 2002	589	0.5	2.8	1–5	10	Mouth
*Clavellotis* sp. from *Coelorinchus kaiyomaru*								
	New Zealand	14 Nov 1983	726	0.5	2	1–4	12	Gill, mouth
*Lernaeopodidae* sp. 1 from *Coelorinchus gilberti*								
	Fukushima	09 Nov 2023	653	0.19	1	1	21	Pelvic fin, anal fin
	Fukushima	14 Nov 2023	496	0	0	0	18	Pelvic fin, anal fin
	Fukushima	14 Nov 2023	533	0.02	2	2	56	Pelvic fin, anal fin
	Fukushima	15 Nov 2023	482	0	0	0	20	Pelvic fin, anal fin
	Fukushima	15 Nov 2023	559	0.05	1	1	20	Pelvic fin, anal fin
Lernaeopodidae sp. 2 from *Coelorinchus gilberti*								
	Fukushima	09 Nov 2023	653	0.23	1.6	1–3	21	Surface (head region)
	Fukushima	14 Nov 2023	496	0	0	0	18	Surface (head region)
	Fukushima	14 Nov 2023	533	0.02	2	2	56	Surface (head region)
	Fukushima	15 Nov 2023	482	0	0	0	20	Surface (head region)
	Fukushima	15 Nov 2023	559	0	0	0	20	Surface (head region)
**Sphyriidae**								
*Lophoura cardusa* from *Hymenocephalus striatissimus*								
	Chiba	22 June 2001	529	0	0	0	16	Front of the dorsal fin
*Lophoura tetraloba* from *Nezumia condylura*								
	Kochi	11 Sep 2002	589	0.14	1.67	1–3	21	Front of the dorsal fin
*Lophoura* sp. from *Coelorinchus gilberti*								
	Fukushima	09 Nov 2023	653	0	0	0	21	Front of the dorsal fin
	Fukushima	14 Nov 2023	496	0.22	1.25	1–2	18	Front of the dorsal fin
	Fukushima	14 Nov 2023	533	0.05	1.33	1–2	56	Front of the dorsal fin
	Fukushima	15 Nov 2023	482	0.05	1	1	20	Front of the dorsal fin
	Fukushima	15 Nov 2023	559	0	0	0	20	Front of the dorsal fin

Of the pennellid species identified, 85% were recorded at depths <500 m. With the exception of *Peniculus, Peniculisa, Exopenna and Parinia* species, the majority of pennellids have a mesoparasitic lifestyle (Kazachenko and Avdeev 1977; Kabata 1979; Boxshall 1986). *Sarcotretes eristaliformis* was recorded at 3600 m, which is the second deepest record among parasitic copepods infecting fish (Figure 2; Kabata and Gusev 1966). These copepods attach to the muscles of host fish, into which their cephalothoracic parts become embedded. Notably, the hosts of deep-sea pennellids are not limited to demersal fish but also included pelagic species (Tables 1, S1 and S2).

Among the sphyriids, 50% were recorded at depths greater than 1000 m (Figure 2). In our specimen survey, only one sphyriid genus, *Lophoura*, was identified (Figure 5C). Sphyriids also exhibit a mesoparasitic lifestyle. The prevalence and intensity of *Lophoura* species were relatively low, with maximum values of 22% and 3.0, respectively, and depth had no apparent influence on these values (Table 1). Similar to pennellids, the cephalothoracic parts of these copepods become embedded within the host muscles or abdominal cavities, mainly from the base of the dorsal fin (as seen in *Lophoura*). The hosts of sphyriids were found to differ according to species and included fish in families Synaphobranchidae and Sebastidae (Tables 1, S1 and S2).

Thirty-seven percent of the deep-sea parasitic copepods were identified as lernaeopodids, with 58% of the species in this family being found at depths <500 m, whereas 15% were recorded at depths >1000 m. This family may have been the most successful in colonizing the deep sea. These copepods are ectoparasites that attach to their hosts by inserting a bulla, an anchoring organ extending from the tip of the maxilla (Figure 5D). Among these species *Parabrachiella annulata* (Markevich 1940), has been established to have the widest distribution and deepest recorded depth (Markevich 1940), with a bathymetric range of 1110–2870 m (Figure 2; Noble 1973; Ho 1975). Among the parasites infecting *Coelorinchus gilberti* Jordan et Hubbs, 1925, gill parasites were found to have a higher prevalence and intensity than those parasitizing the mouth, body surface or fins (Table 1). At almost all survey sites, the prevalence of the gill parasites *Clavella collaris* (Ho 1993) and *Clavella diversia* (Ho 1993), exceeded 50%, reaching up to 95% at some locations. Typically, the hosts of deep-sea lernaeopodids are demersal fish, particularly members of the family Macrouridae, that inhabit considerably deep waters (Tables 1, S1 and S2).

Of the bomolochid species identified, 60% were recorded at depths <500 m. Notably, records of *Hamaticolax resupinus* (Pérez-i-García et al. 2017) extend to 1626 m (Pérez-i-García et al. 2017) (Figure 2; Table S2). By limiting the sampling sites to locations in which parasitic copepods were found and at least 10 host fish were obtained, we found that the prevalence of *Hamaticolax coelorinchi* (Izawa 2022) surpassed 46% at all sites, with a mean intensity >2.0, although ranging widely from 1 to 23. This species was recorded exclusively from Suruga Bay, central Japan, being consistently recovered from 1969 to 2023 (Table 1). Bomolochids were mainly parasitic in the gills and mouths of deep-sea demersal fish (Tables S1 and S2).

Compared with the aforementioned families, records of the families Philichthyidae, Hyponeoidae, Hatschekiidae, Caligidae, Trebiidae, Archidactylinidae, Eudactylindae and Pandaridae were notably scarce, and we were unable to obtain any specimens for these families during our specimen survey. Although only three records were available, caligids appeared to be limited to depths <500 m (Figure 2).

## Discussion

The findings of this study contribute to our understanding of deep-sea parasitic copepods, particularly regarding their bathymetric distribution, prevalence and intensity in the Western Pacific Ocean. For example, the discovery of *Chondracanthodes deflexus* from *Coryphaenoides yaquinae* Iwamoto et Stein, 1974, at a depth of 5171 m represents, to the best of our knowledge, the deepest record to date for copepods parasitizing fish in the Pacific Ocean. Moreover, a literature survey provided insights into global trends in the bathymetric distribution of deep-sea copepods that parasitize fish.

The species richness of deep-sea parasitic copepods on fishes appears to follow the richness of their hosts. In the Pacific Ocean, parasite richness was the highest at approximately 400 m, declined sharply toward approximately 1000 m, and remained low below that depth (Figure 4B). Similarly, the biomass and diversity of pelagic fishes in the Northeastern Pacific Ocean also peak at approximately 400 m and decrease steadily with depth (Smith and Brown 2002). Around Japan, the diversity of Macrouridae peaks between 500 and 599 m before decreasing with depth (Nakayama 2022). In contrast, in the Atlantic Ocean, the species richness of parasites gradually decreases to a depth of 800 m, shows little variation between 800 and 3000 m, and includes only a few species below that depth (Figure 4B). The species richness of deep-sea demersal fish in the Atlantic Ocean is relatively stable between 1000 and 3000 m, although a peak has been identified at approximately 1600 m, which, notably, is characterized by the presence of a permanent thermocline, with Mediterranean overflow water, seasonally strong currents, resuspension of particulate matter, high biomass of benthic filter feeders, and pelagic biomass impinging on the slope (Priede et al. 2010). The species richness patterns of these fishes showed a statistically significant positive correlation with those of parasitic copepods. The observed concordance of species richness between parasites and hosts reflects coevolutionary relationships (Kamiya et al. 2014). We accordingly speculate that our findings in the present study may also be attributable to the coevolution between parasitic copepods and their host fish in the deep sea. Species accumulation curves did not reach a clear plateau at depths of <2000 m, particularly at depths of 200–699 m, indicating that additional sampling might have yielded more species at these depths. In contrast, the curves for deeper depths (>2000 m) tended to saturate at low species richness, suggesting limited species accumulation, even with further sampling. Therefore, although species richness at shallower depths, especially <1000 m, could increase with additional sampling or records, the overall pattern of depth-related species richness is likely to remain unchanged.

Although approximately 65% of the species inhabit depths <500 m, the bathymetric distribution of parasitic copepods tends to be species-specific. The families with representatives recorded at depths exceeding 2000 m are Chondracanthidae, Lernaeopodidae, Pennellidae and Sphyriidae. However, with the exception of Lernaeopodidae, for which four species were reported, for each of these families, only two species were reported at depths exceeding 2000 m. The species within these families constitute a major component of the parasitic copepod community in the deep-sea, most of which parasitize the gills or embed a part of their body into host tissues to directly access a source of nutrients. In deep-water environments, where energy sources are scarce, parasites tend to attach to certain parts of the host body, such as the gills, allowing them to access sufficient nutrients for survival and reproductive success (Hata et al. 2017). Additionally, adult females of species from the four dominant deep-sea families have completely lost their ability to move, conserving energy that would otherwise be used for locomotion and redirecting it toward reproduction. Accordingly, firm attachment to the host is an important factor in expanding the available depth ranges of parasitic species. All families recorded at depths below 2000 m attach to hosts using specific apparatus. For example, chondracanthids utilize their strong uncinate antennae, lernaeopodids deploy a bulla (a modified tip of the maxilla), and pennellids and sphyriids insert their anterior body into host tissues (Kabata 1979; Boxshall and Halsey 2004). These firm attachment methods severely restrict mobility are commonly observed in shallow water, but they may provide stronger selective advantages in the deep sea, where host density is low and energy resources are limited, by ensuring stable attachment or increasing mating efficiency. In contrast, species in the family Caligidae are restricted to the shallowest depth range, below 500 m. To initiate host attachment, caligid species use lunules (specialized cuticular structures) or maxillipeds and antennae, and maintain attachment by employing the cephalothoracic sucker, which entails the expulsion of water from the cephalothorax to generate a negative pressure. This mechanism allows limited movement while remaining attached to the host and facilitates smooth release through slight leg movement (Ohtsuka et al. 2021). However, these mechanisms may be less effective in the high-pressure environments of the deep sea, where generating sufficient negative pressure within the sucker may be challenging.

Additionally, copepod families that include species inhabiting depths >2000 m have unique reproductive characteristics. Notably, chondracanthids, lernaeopodids and sphyriids are characterized by the production of ‘dwarf’ males (Ho 1970; Moran and Piasecki 1994; Piasecki et al. 2006). This trait is often observed in sedentary and sessile species inhabiting environments with low population densities in energy-limited habitats such as the deep sea, where locating mates would be difficult (Ghiselin 1974). Chondracanthids, lernaeopodids and sphyriids are mainly parasitic on demersal fish, which tend to be relatively sedentary and occur at low densities (Boxshall and Halsey 2004). It is possible that the trait evolved in the species parasitic on shallow demersal fish and that this trait became more advantageous in the deep-sea environment. However, although pennellids do not exhibit such a ‘dwarf’ male strategy, some have intermediate hosts, including demersal fish (as in *Lernaeocera* and *Lernaeenicus*) or pelagic gastropod mollusks (as in *Cardiodectes*), particularly among deep-sea species such as *Cardiodectes bellottii* (Richiardi, 1882) (Ho 1966; Perkins 1983; Brooker et al. 2007; Lovy and Friend 2020). By mating on relatively stationary intermediate hosts rather than on highly mobile definitive hosts, parasites can avoid the risk of missing reproductive opportunities. Certain parasites mature and mate while on intermediate hosts, with females departing for definitive hosts after storing sufficient sperm (Brooker et al. 2007). Pennellidae is the only copepod family adapted to parasitize mesopelagic fish (Boxshall 1998), which are strong swimmers, as exemplified by their diel vertical migration behaviour (Pearcy and Laurs 1966; Christiansen et al. 2022). Opportunities for parasites to encounter their hosts are limited, and the contact is often brief. Thus, by completing mating on intermediate hosts, parasites may increase the likelihood of successful infection and reproduction once a definitive host is encountered.

Furthermore, copepods inhabiting areas at depths >2000 m, in which both the density and diversity of host species are low, are typically eurybathic and tend to have low host specificity. The hosts of *Chondracanthodes deflexus* are fish in the families Moridae and Macrouridae (Ho 1985; Boxshall 1998; Kellermanns et al. 2009), whereas those of *Parabrachiella annulata* are fish in the families Macrouridae and Ereuniidae (Markevich 1940; Noble 1973; Ho 1975), and *Sarcotretes eristaliformis* uses species in the families Bathylagidae, Eurypharyngidae, Ipnopidae and Macrouridae (Brian 1908, 1912, 1914, 1929; Wilson 1917; Leigh-Sharpe 1934; Kabata and Gusev 1966). Species in the genus *Lophoura* are regarded as specialists (Boxshall 1998) and the present study indicates that *Lophoura pentaloba* (Ho 1985) exclusively parasitizes species in the family Macrouridae. However, this genus uses a relatively wide range of hosts (two genera each of Macrouridae, *Nezumia* and *Coryphaenoides*) (Ho 1985). Given the necessity for most ectoparasites to actively seek out and attach to hosts, the probability of encountering a specific host is very low in the deep sea. Therefore, having low host specificity may be associated with an increased likelihood of successful attachment. The tendency for deep-sea ectoparasites to have low host specificity may be influenced not only by host availability but also by asymmetries in the respective dispersal capacities of parasites and their hosts. As copepods are characterized by planktonic larval stages and can also disperse among host individuals, it is plausible that parasites can disperse more broadly than their hosts. Thus, having low host specificity may reflect the possibility that parasites do not speciate even when their hosts undergo speciation due to geographical isolation.

The successful colonization of the deep sea by species in certain parasitic copepod families, such as Chondracanthidae, Lernaeopodidae, Pennellidae and Sphyriidae, is assumed to be attributed to both deep-sea colonization of the hosts and the ecology of the parasites. It has been suggested that colonization of the deep sea by fishes increased between 170 and 150 Ma when Pangea began to breakup, thereby providing greater access to the deep sea (Miller et al. 2022). Fossils of cyclopoids parasitic on echinoids date back to approximately 168 Ma (Radwanska and Poirot 2010), and a fossil record of a siphonostomatoid copepod is known from ∼125 Ma (Cressey and Patterson 1973; Cressey and Boxshall 1989). Because both fossil species are considered to be derived species, parasitic copepods are believed to have existed considerably earlier (Bernot et al. 2021). Given these suggestions, parasitic copepods are believed to have diversified prior to the breakup of Pangea, thereby enabling them to follow their hosts into deeper habitats. Among copepods parasitic on fishes, only those associated with hosts that could migrate into the deep sea and have traits conducive to deep-sea survival, such as low host specificity, ‘dwarf’ males, the utilization of intermediate hosts, and firm modes of attachment, might have been able to successfully colonize this extreme environment.

Evidence to date indicates that *Chondracanthodes deflexus* is the only species of parasitic copepod that has successfully expanded its distribution to depths down to 4000 m. This species has been reported from multiple *Coryphaenoides* species. *Coryphaenoides* spp. inhabit a wide depth range and are the most dominant genus within the family Macrouridae at depths >1000 m (Nakayama 2022). Species of *Coryphaenoides* inhabiting abyssal depths form a monophyletic group (Gaither et al. 2016). Chondracanthids are more likely to have co-evolved with their host fish rather than undergone host switching (Paterson and Poulin 1999). Given these insights, the expansion of chondracanthids into the abyssal zone may have occurred along with that of their hosts. Additionally, the prevalence and intensity of *Chondracanthodes deflexus* were relatively high, at 50% and 9.6, respectively. Comparatively, the corresponding values for *Chondracanthodes radiatus* (Müller, 1776), which inhabits waters shallower than those colonized by *C. deflexus*, have been reported to be 2.9% and 1.0 in the Irminger Sea (Palm and Klimpel 2008), and 5.7% and 1.0 in the east Greenland and Irminger seas (Klimpel et al. 2006). In abyssal depths, where fish abundance is low (Bailey et al. 2009), strategies that allow parasitism of the few available hosts such as being parasitic on hosts when they aggregate for feeding or reproduction may be essential (Boxshall 1998).

However, the sphyriid species belonging to the genus *Lophoura* had a relatively low intensity (at most 3.0; Table 1). *Lophoura* species generally insert their bodies into the host tissue near the base of the dorsal fin and have an elongated cephalothorax that facilitates access to the visceral cavity of hosts (Kabata 1979; Ho and Kim 1989). It is possible that this mode of attachment, which imposes a high burden on the host, constrains the number of parasites a single host can tolerate. Similarly, it has been established that the intensity of parasites in which the cephalothorax reaches the hearts of the hosts is limited (Goater and Jepps 2002), which could provide evidence to indicate that the burden of infection may influence parasite intensity. Additionally, the prevalence of some parasitic lernaeopodid species attached to gills was relatively high (>50%), indicating that gills may provide the most beneficial attachment sites for lernaeopodids. Supporting this, 58% of the deep-sea lernaeopodid species and 38% of deep-sea copepods parasitic on fish in our dataset were found attached to gills. The frequent use of gills as an attachment site may be attributed to its selective advantages in deep-sea environments, possibly due to the availability of continuous water flow, which facilitates rich nutrient uptake.

Although among deep-sea copepods, *Hamaticolax coelorinchi* was found to be characterized by a relatively high prevalence (>46%), this was slightly lower than that of shallower-dwelling bomolochid species, such as *Cresseyus confusus* (Stock, 1953), which had a prevalence of 79% off the west coast of Sweden in January 1983 (Höglund and Thulin 1988) and 92% off the North Sea coast of Great Britain (Boxshall 1974). This is presumed to reflect a decline in the density of hosts with increasing depth. In one locality, Suruga Bay, *H. coelorinchi* showed consistent recovery from 1969 to 2023, with a relatively high prevalence (46–76%) and mean intensity (2.84–6.83), which tends to indicate an absence of any marked annual fluctuation during this period (Table 1).

The present study revealed the trends in the bathymetric distribution of copepods parasitic on deep-sea fishes. The species richness of parasites closely mirrored that of their host fishes, suggesting a coevolutionary relationship during the colonization of deep-water environments. Additionally, the findings highlighted that attachment site selection, modes of attachment and reproductive strategies are essential for the survival of parasitic copepods in deep-sea environment. Notably, at depths >2000 m, where host density is low, a eurybathic distribution and low host specificity were observed. At depths >4000 m, where only *Chondracanthodes deflexus* was reported, both prevalence and intensity appeared to be higher.

On the basis of the evidence obtained to date, we speculate that adaptations to the deep-sea environment may have occurred via the following process. Initially, some parasites with deep-sea-adaptive traits, such as ‘dwarf’ males and the use of intermediate hosts to facilitate mate location, as well as modes of firm attachment for efficient energy use, successfully invaded the deep-sea zone by coevolving with their hosts. To further adapt to this low host density environment, these parasites may have adopted additional strategies, including a relaxation of stringent host specificity requirements to enhance the likelihood of encountering hosts, and exhibiting a relatively high prevalence and intensity to maximize their exploitation of the limited available hosts. Over time, parasites lacking such traits may have been selected out in the deep sea, which has low host availability and limited energy resources. In contrast, those possessing or developing strategies such as low host specificity and using host aggregation as a transmission opportunity were better suited to these environments and were thus more likely to continue to survive and progressively proliferate in deeper waters.

During the literature survey, many depth records of parasitic copepods were found missing, even at locations where the host fish were distributed. Some species have only a single depth record, indicating that their potential depth ranges remain unknown. Thus, the accumulation of bathymetric data is crucial for a more detailed analysis, including comparisons of species richness between the Pacific and Atlantic Oceans. Furthermore, molecular phylogenetic analyses may help clarify the pathways of expansion into the deep sea, including the timing of colonization, while considering both the history of host colonization and the paleoenvironment of the deep sea.

## Supporting information

Yumura et al. supplementary material 1 Yumura et al. supplementary material

Yumura et al. supplementary material 2 Yumura et al. supplementary material

Yumura et al. supplementary material 3 Yumura et al. supplementary material

Yumura et al. supplementary material 4 Yumura et al. supplementary material

## Supplementary material

The supplementary material for this article can be found at https://doi.org/10.1017/S0031182025100851.
