# Supplementary material for: Bathymetric distribution of parasitic copepods: strategies for adaptation to the deep-sea environment
Source: Parasitology. 2025 Sep 19;152(12):1263–74. doi: 10.1017/S0031182025100851 (PMC12921265; doi:10.1017/S0031182025100851)
Supplement: Yumura et al. supplementary material 1 — Yumura et al. supplementary material [file S0031182025100851sup001.docx]

**Supplementary Information 1.** Species used in Figure 2 are listed below in sequence from the left.

**Chondracanthidae**: *Chondracanthodes deflexus, Lateracanthus quadripedis*, *Diocus frigidus*, *Chondracanthus cottunculi*, *Chelonichondria okamurai*, *Lateracanthus novus*, *Chondracanthodes radiatus*, *Jusheyhoea moseri*, *Chondracanthus dibranchi*, *Chondracanthus neali*, *Jusheyhoea ryukyuensis*, *Scheherazade scheherazade*, *Chondracanthodes tuberofurcatus*, *Chondracanthus yanezi*, *Acanthochondria lepidionis*, *Acanthochondria incisa*, *Chondracanthus genypteri*, *Rohdea cryptopoda*, *Chondracathus polymixiae*, *Chondracanthus colligens*, *Jusheyhoea macrura*, *Diocus semilunaris*, *Chondracanthus pinguis*, *Chondracanthus triventricosus*, *Acanthochondria bicornis*, *Humphreysia floreata*, *Chondracanthus lophii*, *Immanthe campanulata*, *Avatar nishidai*, *Kokeshioides surugaensis*. **Penneiildae:** *Sarcotretes eristaliformis*, *Exopenna crimmeni*, *Cardiodectes bellottii*, *Sarcotretes scopeli*, *Peniculus hokutoae*, *Haemobaphes cyclopterina*, *Protosarcotretes nishikawai*, *Protosarcotretes multilobatus*, *Haemobaphes diceraus*, *Peniculus clavatus*, *Sarcotretes umitakae*, *Protosarcotretes gnavus*, *Phrixocephalus* sp. **Sphyriidae:** *Lophoura pentaloba*, *Lophoura simplex*, *Periplexis lobodes*, *Lophoura tetraphylla*, *Sphyrion lumpi*, *Lophoura bouvieri*, *Lophoura gracilis*, *Lophoura szidati*, *Lophoura tetraloba*, *Lophoura cornuta*, *Lophoura edwardsi*, *Lophoura* sp., *Lophoura cardusa*, *Lophoura unilobulata.* **Lernaeopodidae:** *Parabrachiella annulata*, *Naobranchia maxima*, *Nudiclavella galapagoensis*, *Lernaeopodina spinacis*, *Parabrachiella pinguis*, *Kabatahoia pectinata*, *Lernaeopodina longibrachia*, *Clavella adunca*, *Clavella sokodara*, *Clavellotis* sp., *Parabrachiella nitida*, *Neoalbionella longicaudata*, *Clavella deminuta*, *Vanbenedenia hydrolagae*, *Clavella collaris*, *Clavella diversia*, *Lernaeopodidae* sp.1, *Lernaeopodidae* sp.2, *Clavella* sp.4, *Clavellomimus macruri*, *Clavella* sp.2, *Clavella* sp.1, *Clavella uncinata*, *Nectobrachia producta*, *Praeclavella gracilis*, *Clavella gadomi*, *Clavella* sp.5, *Naobranchia* sp.2, *Parabrachiella superba*, *Praeclavella ovata*, *Clavella tumidula*, *Cryptova limbifera*, *Naobranchia alta*, *Parabrachiella brevibrachiata*, *Parabrachiella chlorophthalmi*, *Parabrachiella microdigitata*, *Parabrachiella mirifica*, *Neoalbionella etmopteri*, *Naobranchia* sp.1, *Praeclavella stichaei*, *Clavella perfida*, *Eubrachiella antarctica*, *Eubrachiella gaini dorsituberculata*, *Eubrachiella gaini gaini*, *Praeclavella fortis*, *Praeclavella singularis*, *Clavella* sp.3, *Vanbenedenia kroeyeri*, *Clavellopsis pellucidula*, *Clavella* sp.6, *Praeclavella parva*, *Parabrachiella robusta*, *Neoalbionella centroscyllii*, *Anaclavella filifera*. **Eudactylinidae:** *Heterocladius abyssetes*, *Protodactylina pamelae*, *Eudactylina acanthii*. **Philichthyidae:** *Sarcotaces antimori*, *Sarcotaces komaii*, *Sarcotaces namibiensis*, *Sarcotaces arcticus*, *Procolobomatus kyphosus*. **Trebiidae:** *Trebius bilobatus*. **Bomolochidae:** *Hamaticolax resupinus*, *Hamaticolax juanji*, *Hamaticolax coelorinchi*, *Hamaticolax physiculi*, *Parahamaticolax ezoisoainame*. **Hyponeoidae:** *Tautochondria dolichoura*. **Hatschekiidae:** *Laminohatschekia synaphobranchi*, *Brachihatschekia lobulata*, *Prohatschekia stocki*, *Hatschekia etelisicola*, *Hatschekia fusiformis*, *Prohatschekia awatati*, *Hatschekia hoplobrotulae*, *Prohatschekia neobythitesi*, *Hatschekia squamata*. **Pandaridae:** *Demoleus heptapus*, *Echthrogaleus denticulatus*, *Pagina tunica*. **Caligidae:** *Caligus cresseyorum*, *Lepeophtheirus hippoglossi*, *Caligus rapax*. **Archidactylinidae:** *Archidactylina myxinicola*.
